# Simplified Synthesis of Poly(ethyleneimine)-Modified Silica Particles and Their Application in Oligosaccharide Isolation Methods

**DOI:** 10.3390/ijms25179465

**Published:** 2024-08-30

**Authors:** Xingyun Zhao, Yifan Niu, Chengxiao Zhao, Zhenyu Li, Ke Li, Xuemei Qin

**Affiliations:** Modern Research Center for Traditional Chinese Medicine, The Key Laboratory of Chemical Biology and Molecular Engineering of Ministry of Education, Key Laboratory of Effective Substances Research and Utilization in TCM of Shanxi Province, Shanxi University, No. 92, Wucheng Road, Taiyuan 030006, China; 202224301007@email.sxu.edu.cn (Y.N.); 202224302038@email.sxu.edu.cn (C.Z.); lizhenyu@sxu.edu.cn (Z.L.); qinxm@sxu.edu.cn (X.Q.)

**Keywords:** nucleophilic substitution, epoxy ring-opening reaction, HILIC stationary phase, polar compound, TCM oligosaccharides

## Abstract

There are great challenges in the field of natural product isolation and purification and in the pharmacological study of oligosaccharide monomers. And these isolation and purification processes are still universal problems in the study of natural products (NPs), traditional Chinese medicine (TCM), omics, etc. The same polymer-modified materials designed for the special separation of oligosaccharides, named Sil-epoxy-PEI and Sil-chloropropyl-PEI, were synthesized via two different methods and characterized by scanning electron microscopy combined with energy spectrum analysis, Fourier transform infrared spectroscopy, thermogravimetric analysis, zeta potential as well as surface area analysis, etc. Several nucleotide/nucleoside molecules with different polarities and selectivities were successfully isolated in our laboratory using stainless-steel columns filled with the synthesized material. In addition, the separation of saccharide probes and oligosaccharides mixtures in water extracts of Morinda officinalis were compared in HILIC mode. The results showed that the resolution of separations for the representative analytes of the Sil-epoxy-PEI column was higher than for the Sil-chloropropyl-PEI column, and the developed stationary phase exhibited improved performance compared to hydrothermal carbon, amide columns and other HILIC materials previously reported.

## 1. Introduction

Studies indicate that oligosaccharides obtained by extraction from natural sources possess pharmacological effects such as enhanced immunity, antibiotic effects, and antioxidant effects [1,2]. And isolation of saccharides’ active compounds in the study of natural products is a great challenge [3]. Generally, methods for the separation of oligosaccharides are reported in many works, and include (but are not limited to) chromatography [4], capillary zone electrophoresis [5] and membrane separation [6]. There are many types of chromatography, including gel exclusion chromatography [7], hydrophilic interaction chromatography [8], anion exchange chromatography [9], graphitized carbon column chromatography [10], reverse-phase high-performance liquid phase chromatography [11], etc. Chromatography is a proven and a popular method for the separation and preparation of oligosaccharides. According to previous research, the most common approach used for sugar analysis is high-performance liquid chromatography (HPLC) [12]. Many traditional HPLC analytic systems that include plenty of functional groups, namely zwitterion [13,14], carbohydrate [15,16], amide/amine [17,18], MOF/COF [19,20] and other types, have been described with both good selectivity and separation ability. Click saccharide stationary phases, such as glucose, maltose, and cyclodextrins bonded to silica gel surfaces, were proven to be effective separation materials for hydrophilic interaction liquid chromatography for oligosaccharide analysis [21,22]. 

Moreover, in related works, carbon derived from cyclodextrin-modified silica (Silica@HTC) stationary phases created by one-pot hydrothermal functionalization modification showed an efficient separation of polar saccharides or glycans compounds, making use of their hydrophilic characteristics [23,24,25]. Furthermore, there were attempts to introduce polyacrylamide (PAM) [26,27,28], polyvinylpyrrolidone (PVP) [29], polyvinyl alcohol–cellulose [30] chitosan [31] and so on onto silica surfaces as a HILIC stationary phase or as advanced materials for oligosaccharides. However, separation of saccharides is still a relevant and difficult scientific issue. 

During the past few years, the amino column has been considered as a gold-standard column for sugar separation due to amino groups. Polyethyleneimine (PEI) is composed of primary amine, secondary amine, tertiary amine and other groups and is endowed with unique properties and high chemical reaction activity; specifically, these functional groups can be further modified by grafting on other active groups with better properties [31,32]. Hence, a range of applications including wastewater treatment (WWT), applications to gene carriers, sustained drug release strategies, etc., have been proposed and formed [33,34]. Additionally, PEI with rich amino groups is conjectured to have the capability of providing anion exchange and hydrophilic interactions in the field of stationary phases [35]. Therefore, PEI has widespread applications in HPLC stationary phases for protein and nucleosides, polar organics and peptides, etc. For example, PEI molecules, which are reported to adsorb onto silica particles via electrostatic force between PEI and a negatively charged silica surface, are unstable and bleed at low pH due to the very weak acid properties of silanol groups [36]. Several proteins were isolated with good efficiency and recovery using an adsorbed PEI-bonded phase, which was synthesized by adsorption and cross-linking PEI 200 onto the fused-silica capillary inner wall [37]. In another example, in 2014, poly (HPMA-Cl-co-EDMA) microspheres had PEI grafted onto them, making use of a nucleophilic substitution reaction [38], and they were then poured into microbore columns and used in the hydrophilic interaction chromatography (HILIC) stationary phase for the successful separation of polar compounds such as nucleosides, peptides, etc. More impressively, poly (ethylene terephthalate) (PET) was modified using PEI and cross-linked by a 1,4-butanediol diglycidyl ether (BUDGE), namely a PET-PEI/BUDGE stationary phase. This phase has been previously applied for protein separations, as it is superior to PEI-modified monoliths [39]. However, there have not yet been systematic comparative studies on the separation performance or molecular interaction between PEI and active oligosaccharides and the stability of these oligosaccharides and it is essential to develop a stable and efficient PEI-immobilized silica stationary phase.

Inspired by the aforementioned studies, in this paper, a synthesized HILIC stationary phase for saccharides analysis was researched and contrasted via two reaction methods created by grafting PEI onto the surface of silica spheres called Sil-epoxy-PEI and Sil-chloropropyl-PEI. HPLC analysis was carried out to verify the separation ability of the bonded phase by testing slightly hydrophilic aniline compounds, nucleoside compounds and saccharides in HILIC mode. In addition, the HILIC mode of the stationary phase was compared by the analysis of oligosaccharides in samples of natural product extract complexes. The result shows that this method was successfully applied to separate natural products or TCMs oligosaccharides. Remarkably, the experimental results showed fine specificity and enormous separation potential for oligosaccharides on the Sil-epoxy-PEI column compared to the Sil-chloropropyl-PEI column.

## 2. Results and Discussion

### 2.1. Physical and Chemical Analysis Test of the Sil-Epoxy-PEI and Sil-Chloropropyl-PEI Materials 

**Figure 1:**
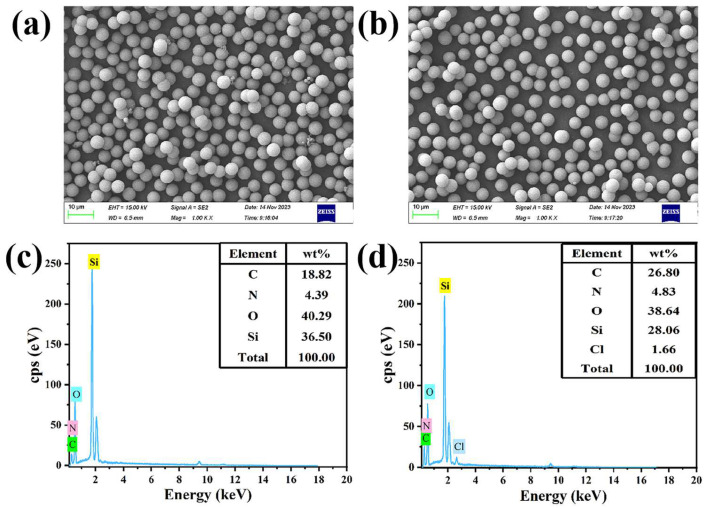
Scanning electron micrographs and the corresponding EDS of Sil-epoxy-PEI packing particles (**a**,**c**) and Sil-chloropropyl-PEI packing particles (**b**,**d**).

The prepared Sil-epoxy-PEI and Sil-chloropropyl-PEI were first characterized by scanning electron microscopy (SEM) and energy-dispersive spectroscopy (EDS). The morphology of Sil-epoxy-PEI and Sil-chloropropyl-PEI, captured by scanning electron microscopy, are displayed in Figure 1a,b, showing that the homogeneity and spherical shape of the two modified materials were well maintained. Then, 5 kV of the scanning electron microscope was used to examine the surface composition. Energy-dispersive X-ray spectroscopy (EDX, EDS) provides a quantitative and/or qualitative identification of chemical elements in the entirety of nano/micromaterials. After electron beam bombardment of the sample, emitted X-ray intensity is measured in the function of the applied energy. The depth of analysis can nevertheless reach 1 μm; this is generally associated with electron microscopy techniques such as SEM or TEM. Information on elemental composition, including contents of carbon, oxygen, S, Si, N, P, etc., can be obtained from energy-dispersive X-ray spectroscopy analysis. After image acquisition and EDX analysis, the results of the EDS data showed that the wt.% of Cl on the surface of Sil-chloropropyl-PEI spheres was 1.66%, while no Cl was found on the surface of Sil-epoxy-PEI. From the EDS data, it was found that the wt.% C was 26.8% and wt.% N was 4.83% on the Sil-chloropropyl-PEI spheres’ surface (Figure 1d). However, it was concluded that the wt.% N and C on the Sil-epoxy-PEI spheres’ surface was about 18.82% and 4.39% (Figure 1c). This indicates the presence of a relatively large amount Si and O on the Sil-epoxy-PEI surface. PEI was reacted with an epoxy silanizing agent/chloropropyl trichlorosilane separately, and the resulting compounds were analyzed by nuclear magnetic resonance spectroscopy, which proved that the structures differed, and the results indicate that the reaction was successful (see Figure S1).

**Figure 2:**
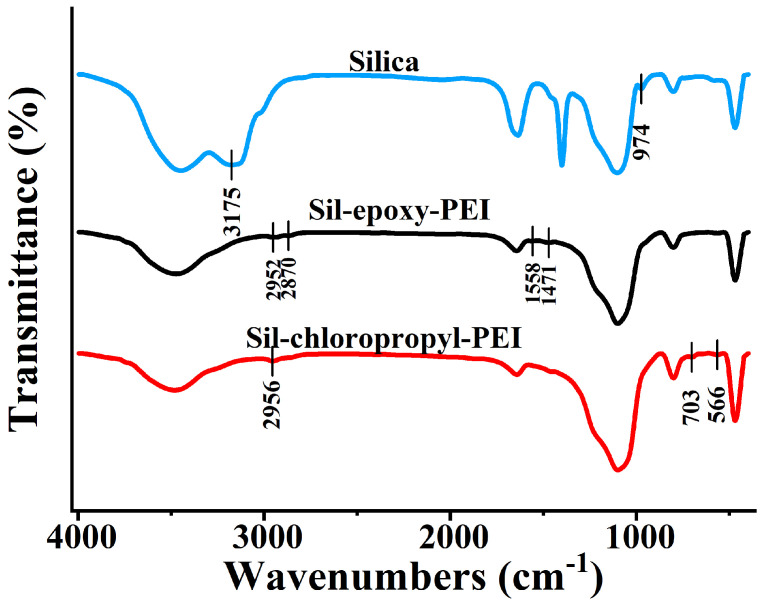
FT−IR spectra of the Silica (blue line), Sil-epoxy-PEI packing particles (black line) and Sil-chloropropyl-PEI (red line).

The IR characterization was also demonstrated in Figure 2, in which the bands at 2952 and 2870 cm^−1^ were assigned to stretching vibrations of C-H in the methylene groups. Peaks at 1558 and 1471 cm^−1^ of the synthesized materials were attributed to bending vibrations of O-H and N-H. Compared with the infrared spectrum of silica, the stretching vibration peak of silica hydroxyl group 3455 cm^−1^ and 974 cm^−1^ decreased on modified silica. In addition, the characteristic peak of amino groups was at around 3481 cm^−1^, indicating that PEI was successfully grafted onto the surface of both synthesized materials. Furthermore, the absorption peak at 1333 cm^−1^ was possibly due to the stretching vibration of C-N [40,41]. Besides, the pore structure was tested and showed in Figure S2 and Table S1. The zeta potential assay of the Sil-epoxy-PEI packings (black line) and Sil-chloropropyl-PEI packings (red line) is pictured in Figure S3. The charges became positive on the Sil-epoxy-PEI and Sil-chloropropyl-PEI surfaces, in contrast to the negative charge of the silica surface.

**Figure 3:**
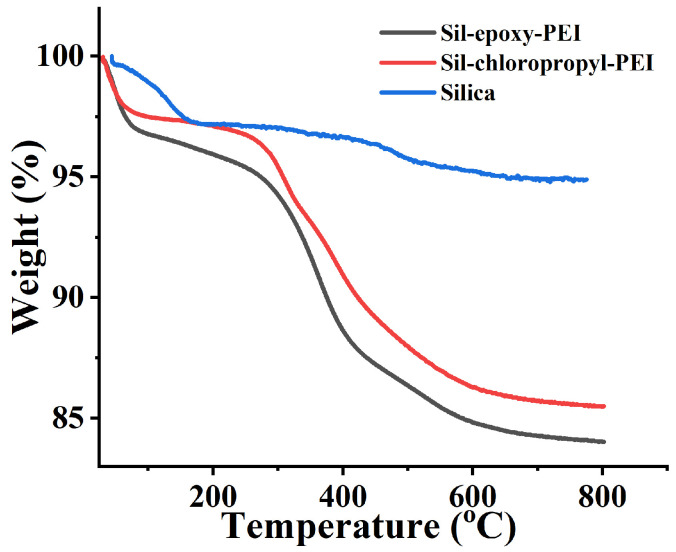
TGA curves of the original silica (blue line), Sil-epoxy-PEI packings (black line) and Sil-chloropropyl-PEI spheres (red line).

The thermal stability and the number of organic functional groups of the synthesized materials (Silica, Sil-epoxy-PEI, and Sil-chloropropyl-PEI) were evaluated through thermogravimetric analysis with the temperature ranging from 30 °C to 800 °C. The percentage of functional groups modified on silica can be estimated based on the mass loss of the samples at high temperatures, which is shown in Figure 3. Sil-epoxy-PEI exhibited a mass loss rate of approximately 16% as the temperature rose due to the loss of organic fractions. When the temperature arrived at 120 °C, the mass of both materials decreased to 96% and 97%, possibly due to the adsorptive water. As the temperature further increased to 350 °C, the mass decreased sharply to 86% on Sil-epoxy-PEI, while the mass only decreased to 93% at 350 °C on Sil-chloropropyl-PEI materials, respectively. When the temperature was 800 °C, the total mass loss was 16% and 15%, respectively. The results of the TGA analysis were in agreement with the IR data. The above thermogravimetric changes implied that the target groups were successfully modified on the silica. After processing the above data, it was clearly proven that the stationary phase material had been successfully synthesized.

### 2.2. Chromatographic Evaluation of the Two Prepared Columns

#### 2.2.1. Stability Evaluation of Batch-to-Batch Reproducibility

For a newly developed analytical or isolation method, reproducibility from run to run and batch to batch is an important evaluation parameter. Three batches of separation materials were synthesized under the same operating steps; polar probes of nucleotides, bases, and saccharides were chosen for run-to-run and batch-to-batch stability. Run-to-run results from different days or a few weeks apart were calculated as 1.1%. The deviation of retention time for these compounds on three batches of columns varied between 0.04 and 0.3 min where the maximum value of RSD was 4% under the same chromatographic conditions, indicating good batch-to-batch column reproducibility (Figure S4 and Table S2).

#### 2.2.2. Chromatographic Separation Test of Nucleosides and Nucleoside Bases under HILIC Mode

**Figure 4:**
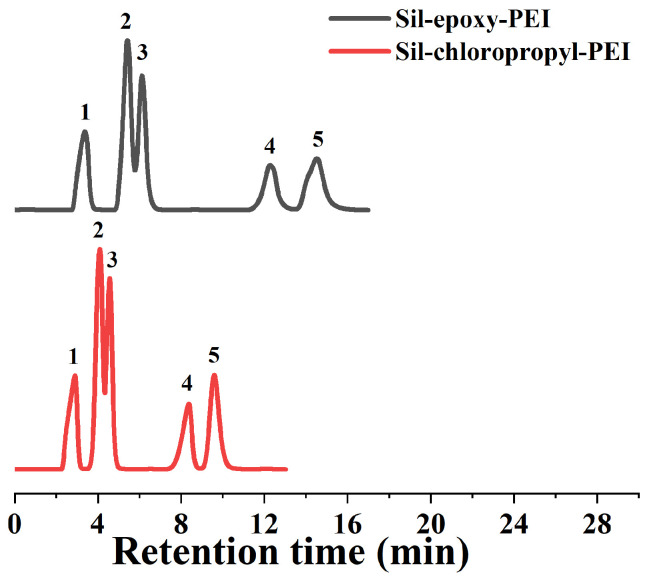
Separation exploration of nucleosides and nucleoside bases on both synthesized Sil-epoxy-PEI (black line) and Sil-chloropropyl-PEI (red line). Experimental conditions: 4.6 mm i.d ×150 mm length, mobile phase: ACN/water (85/15, *v*/*v*), flow rate: 1 mL/min, peaks: (1) β-Thymidine; (2) adenosine; (3) uridine; (4) cytidine; (5) inosine.

To verify the character of the hydrophilic polymer stationary phase, the separation of nucleosides was generally performed in HILIC mode. Thus, polar test mixtures consisting of beta-thymidine, adenosine, guanosine, cytidine, and inosine were selected as the model compounds, which were also separated on two columns and a commercial amino column under the same HILIC conditions. As shown in Figure 4, for the Sil-epoxy-PEI column, five compounds are conspicuously separated with the isocratic eluting mobile phase ACN content at 85%. The detailed chromatography data are given in Supplementary Table S2. In contrast, five compounds are, at baseline, separated by shorter time spans on the Sil-chloropropyl-PEI column (CAN = 85%). The column efficiency of these four compounds was between 40,000 and 60,000 m^−1^. Meanwhile, anilines which belonged to moderately polar compounds with weak alkaline were always applied to chromatographic separation comparation experiments, and the results are shown in Figure S5. It was proven that the separation ability of these kinds of polar compounds is more feasible compared to that reported in some studies. In addition, we proved that the resulting polymer-modified particles (Sil-epoxy-PEI) via the ring-opening polymerization of 3-glycidoxypropyltrimethoxysilane had relatively strong hydrophilicity.

### 2.3. Chromatographic Separation Comparison of Standard Saccharides Compounds under HILIC Mode

**Figure 5:**
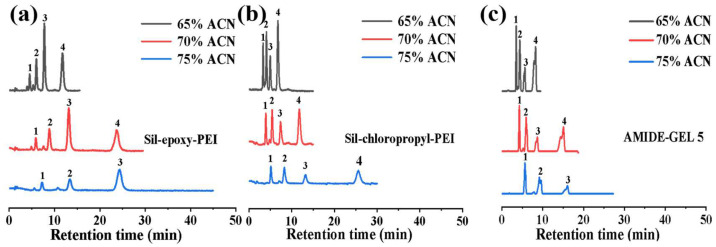
Separation of saccharide compounds with varying concentrations of acetonitrile in mobile phase on Sil-epoxy-PEI packing particles column (**a**), Sil-chloropropyl-PEI packing particles column (**b**) and AMIDE-GEL 5 (**c**). Analytes: (1) glucose, (2) maltose, (3) raffinose, (4) stachyose; experimental conditions: 15 cm length × 4.6 mm i.d. mobile phase method: ACN/water, 65% ACN (black lines), 70% ACN (red lines), 75% ACN (blue lines). flow rate: 1 mL/min.

It is highly desirable to isolate and purify oligosaccharides from water extracts of NPs or traditional Chinese medicines. Hence, four non-reducing sugars including mono-, di-, tri-, and tetra saccharides were chosen to be model sugars. Baseline separation was obtained, and their elution order was in accordance with the sugar’s polarity from weak to strong, which was consistent with the typical retention mechanism of HILIC. These typical examples clearly showed good chromatograms of the three columns, which were successively eluted with 65%, 70%, and 75% acetonitrile content isocratic elution. The elution fraction was then connected and detected by HILIC–ELSD. As can be seen from Figure 5 and Table S3, eluates showed well-separated peaks; for raffinose, the retention time was increased from 4.9 min to 13.2 min on Sil-chloropropyl-PEI, while for the Sil-epoxy-PEI column, the retention time was prolonged from about 7.7 min to 24.2 min. The retention time of raffinose was only increased from 5.6 min to 16.06 min on the commonly used commodity amide column, which had proven effective for performing sugar analysis. One possible reason for this is that there are hydrogen bond interactions between hydroxy groups, oxygen, and carbohydrate molecules on the Sil-epoxy-PEI column. Overall, the results of high retention time and high resolution of separation suggest that it is a beneficial method for carbohydrate analysis.

### 2.4. Chromatographic Separation of NPssaccharides Complex Samples under HILIC Mode

**Figure 6:**
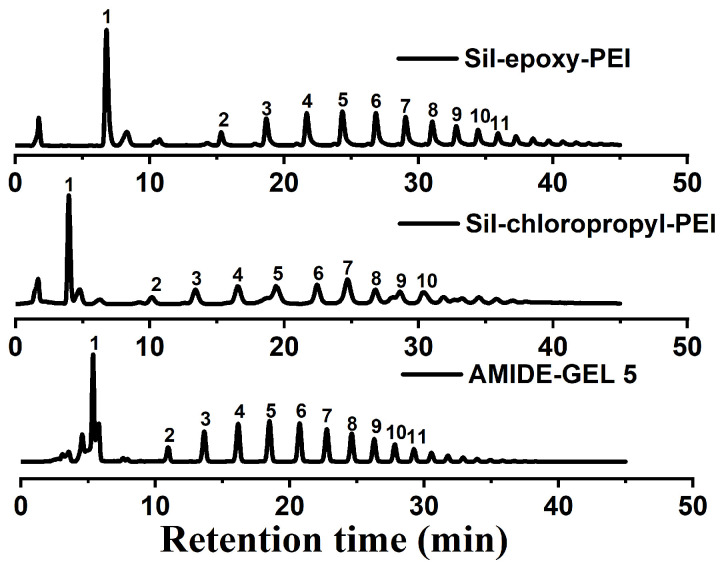
Results of separation of saccharides from water extract of Morinda officinalis on Sil-epoxy-PEI made in the research group, Sil-chloropropyl-PEI made in the research group and the purchased AMIDE-Gel 5; mobile phase gradient: A-water, B-ACN, gradient elution: 0~30 min, 25%~50% A, 75%~50% B, 30~50 min, 50% A, 50% B, peak info: the peak number in figure represents polymerization degree value of oligosaccharide, flow rate: 1 mL/min. Detector: evaporative light scattering, tube temperature: 50 °C.

In this part, mainly comparative work was performed to determine the isolation applicability of the Sil-epoxy-PEI and Sil-chloropropyl-PEI column for the separation of compounds in NPs/TCM water extracts. The results clearly exhibit the utility of Sil-epoxy-PEI for the efficient separation of saccharides, as shown in Figure 6. Excellent peak shape and resolution for four model compounds was observed by the Sil-epoxy-PEI column and high separation efficiency (e.g., ~52,000 m^−1^ for tetrose) was achieved as well. By contrast, the amide column and Sil-chloropropyl-PEI column demonstrated less retention for these compounds in which the chromatographic peak separation resolution was lower than PEI materials. It was concluded that retention ability of saccharides on Sil-epoxy-PEI was better than saccharides on the commercial amide column and Sil-chloropropyl-PEI column (shown in Figure 6). Detailed data can be found in Table S4. And more other columns synthesized in our own lab such as Sil-vinyl-GSH (the chromatography is displayed in Figure S6), Sil-vinyl-acrylamide column (the chromatography graph is shown in Figure S7) and silica@HTC (the graph is shown in Figure S8) were used for the separation of a mixture of typical sugar polar compounds connected with evaporative light scattering detection (ELSD) under the same conditions. Therefore, the Sil-epoxy-PEI column has great application potential for saccharides, among all of these. One possible reason is the presence of numerous amino groups in both of them, especially the presence of many hydroxy groups by ring-opening polymerization on Sil-epoxy-PEI. Thus, this work is meaningful and provides a foundation for the subsequent modification of the functional groups used in the separation and analysis of natural products or traditional Chinese medicine oligosaccharides. It is beneficial for the future study of pharmacological effects.

## 3. Conclusions

In this study, two different stationary phase particles were successfully synthesized via two methods, which were compared for isolation performance as well as wide-range retention characteristics specifically for oligosaccharides. In summary, hydrophilic interaction modes for nucleosides and standard saccharides or aniline compounds were efficiently evaluated on both columns. In addition, we demonstrated the capacity to separate the carbohydrates in NPs by using two different materials that were synthesized either via the ring-opening polymerization of 3-glycidoxypropyltrimethoxysilane and poly(ethylenimine) or the nucleophilic substitution of 3-chloropropyl trichlorosilane and poly(ethylenimine). The unique surface physical and chemical properties make the Sil-epoxy–PEI material a versatile stationary phase, not only for the chromatographic separation of saccharides, but with many potential applications to be researched via various post-modifications. In the future, more relevant studies regarding separation materials of higher column efficiency and amplifying the preparation of polar chemical components of natural medicines are expected.

## 4. Experimental Section and Reagents and Materials

Spherical porous silica (size 5 μm, pore 9.5 nm) was purchased by Prof. Shudong Wang’s Group from the Dalian Institute of Chemical Physics, Chinese Academy of Sciences, Dalian, China. The epoxy 3-glycidyl ether oxy propyl trimethoxy-silane (epoxy as functional group), chloropropyl trichlorosilane (chloropropyl as functional group), and polyethyleneimine (PEI, Mw = 300) were obtained from Sigma-Aldrich (St. Louis, MO, USA). β-thymidine, adenosine, uridine, cytidine, inosine, saccharides were purchased from Aladdin (Shanghai, China). All the benzylamine, p-phenylenediamine, o-phenylenediamine were purchased from Sigma–Aldrich (St. Louis, MO, USA). Toluene, methanol, and ethanol were from Damao chemistry (Tianjing, China). Stainless-steel empty columns (4.6 mm i.d.× 150 mm length) were purchased from Weida Analytical Instrument Factory (Dalian, China). HPLC-grade ACN and pure water was used for the preparation of mobile phases. The water used in all experiments was doubly distilled and purified by Milli-Q system (Millipore Inc., Milford, MA, USA) unless otherwise stated. Other reagents were routinely obtained as received.

### 4.1. Instrumentation

Scanning electron microscopy (SEM) images were obtained using the Zeiss Sigma 300 (Zeiss AG, Oberkochen, Germany). Fourier transform infrared spectroscopy (FT-IR) characterization experiments were carried out with 2–5 mg materials using a Bruker FTIS Tensor 27 spectrometer (Bruker, Karlsruhe, Germany). The LC experiments were performed using an HPLC instrument composed of CTO-20A column oven, LC-20AT two pumps, a UV detector SHIMADZU SPD-20A, and evaporative light scattering detection (ELSD) (Shimadzu Enterprise Management, Tokyo, Japan). The retention factor (k′) was calculated as follows:

$$k^{\prime }=\left(t_{r}-t_{0}\right)/t_{0}$$

where t_r_ and t_0_ are the retention time of compounds and unretained compounds, respectively. The resolution of two compounds (Rs) was calculated by the following equation:

$$R_{s}=2\left(t_{R2}-t_{R1}\right)/\left(W_{b2}+W_{b1}\right)$$

And the column efficiency was calculated via N/m:

$$16\times {\left(\frac{\mathrm{tr}}{w}\right)}^{2}\times (\frac{100}{L})\;$$

in which formulas tr, w and L are denoted as the retention time of probes, peak width and the column length, respectively.

### 4.2. Synthesis of Sil-Epoxy-PEI and Sil-Chloropropyl-PEI

#### 4.2.1. Activation and Functional Modification

The silica particles were activated and prepared with reference to the previous article from Wang’s group [42]. The silica microspheres were refluxed in concentrated hydrochloric acid and nitric acid aqueous solution as described above. Then, dried activated silica (2.10 g) was added to a round-bottom flask with solvent such as anhydrous toluene (60 mL) and 2.2 mL chloropropyl trichlorosilane or epoxy silanizing reagent (2.2 mL), which was fabricated at 115 °C for more than 20 h for comparation.

#### 4.2.2. Synthesis of Sil-Epoxy-PEI, Sil-Chloropropyl-PEI and Other HILIC Particles

**Figure 7:**
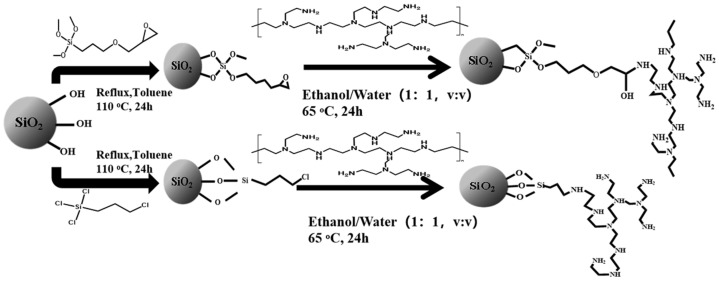
Schematic diagram of the preparation route of the Sil-epoxy-PEI and Sil-chloropropyl-PEI stationary phase.

As was displayed in Figure 7, the synthetic method for producing Sil-epoxy-PEI and Sil-chloropropyl-PEI was similar to the method described by Ren et al., with a slight change [43]. The obtained materials and 2 mL PEI mixtures were dispersed in 70 mL ethanol/water (1:1, *v*/*v*) and stirred at 60 °C for 24 h. Then, the obtained materials were filtered and washed with water and anhydrous ethanol again. Finally, the prepared product with which the functional groups were reacted and grafted was simply obtained after drying under a vacuum. The above functional silica of Sil-chloropropyl and PEI were also suspended in 70 mL ethanol/water (1:1, *v*/*v*) at the same time. The mixture was stirred at 60 °C for 24 h. The solid matter was filtered and washed with water and 95% anhydrous ethanol. Also, the products were mixed with glucose solution for a while and obtained after washing and drying under a vacuum for a set amount of time.

The preparation process of other materials and the isolation method comparison are shown in the Supplementary Materials.

#### 4.2.3. Column Packing Method

All of the prepared materials (2.3 g), including Sil-epoxy-PEI and Sil-chloropropyl-PEI in this study, and Sil-vinyl-GSH, Sil-vinyl-acrylamide, and Silica@HTC in previous studies, were mixed with 20 mL methanol/isopropanol and poured into a 25 mL homogenate tank. The air compressor was started with the methanol that was used as the driving agent, immediately regulated to 30 MPa for 5–10 min, again increased to 40 MPa for 5 min, and then the pressure was then allowed to decrease to zero. It was taken down after standing for 10 min in the column and washed with ACN/water for 3 h before the HPLC chromatographic analysis.

## Data Availability

The data supporting this article have been included as part of the Supplementary Materials.

## Supplementary Materials

The following supporting information can be downloaded at https://www.mdpi.com/article/10.3390/ijms25179465/s1 [44].

## Abbreviations

HPLC	High-performance liquid chromatography
NPs	Natural products
TCM	Traditional Chinese medicine
HILIC	Hydrophilic interaction liquid chromatography
ELSD	Evaporative Light Scattering Detection
HTC	Hydrothermal carbon
TGA	Thermogravimetric analyzer
FT-IR	Fourier transform infrared spectroscopy
GSH	Glutathione
EDS or EDX	Energy-dispersive spectroscopy
