# Supplementary material for: Simplified Synthesis of Poly(ethyleneimine)-Modified Silica Particles and Their Application in Oligosaccharide Isolation Methods
Source: Int J Mol Sci. 2024 Aug 30;25(17):9465. doi: 10.3390/ijms25179465 (PMC11395661; doi:10.3390/ijms25179465)
Supplement: Supplementary file 1 [file ijms-25-09465-s001.zip › ijms-3155802-supplementary.pdf]

Supporting Information

Simplified Synthesis of the Poly(Ethyleneimine) Modified Silica Particles and Their Application in Oligosaccharides Isolation Method

Xingyun Zhao[1,2,3*], Yifan Niu[1,2,3], Chengxiao Zhao[1,2,3], Zhenyu Li[1,2,3], Ke Li[1,2,3*], Xuemei Qin[1,2,3]

[1] Modern Research Center for Traditional Chinese Medicine, Shanxi University, No. 92, Wucheng Road
Taiyuan, 030006, Shanxi, P. R. China
[2] The Key Laboratory of Chemical Biology and Molecular Engineering of Ministry of Education, Shanxi University, No. 92, Wucheng Road
Taiyuan, 030006, Shanxi, P. R. China
[3] Key Laboratory of Effective Substances Research and Utilization in TCM of Shanxi Province
Shanxi University, No. 92, Wucheng Road
Taiyuan, 030006, Shanxi, P. R. China
E-mail: like@sxu.edu.cn, xyzhao@sxu.edu.cn
* Correspondence: el: +86-411-7018379, ORCID:0000-0002-3596-6820
E-mail address: like@sxu.edu.cn, xyzhao@sxu.edu.cn

[*]Corresponding Author. Tel: +86-411-7018379，ORCID：0000-0002-3596-6820

E-mail address: like@sxu.edu.cn, xyzhao@sxu.edu.cn

This work is a stage research summary of scientific issues which is related to the separation and purification of traditional Chinese medicine/Natural products oligosaccharides. At the same time, a series of reports by the research group is on the way on the core media in the field of analytical chemistry, especially chromatographic analysis and the specific isolation and purification oligosaccharides in traditional Chinese medicine/natural medicine extracts making use of modified macroporous resin or silica media.

Preparation method of the other materials

The preparation methods of one of the hydrophilic materials (Sil-vinyl-GSH). The Sil-vinyl-GSH was similar to the Ref [1]. The detail process is as followings: 2.3 g of vinyl functionalized silica was added to 50 ml of methanol/water (7:5, v:v), ultrasonic, then 3.0g of glutathione was added and stired at room temperature and then the temperature was raised to 70 °C, 100 mg AIBN was also added and stirring the reaction for 6 h, and the centrifugation products were washed with water and methanol in order to obtain the Sil-vinyl-GSH.

The preparation methods of one of the hydrophilic particles (Sil-vinyl-acrylamide): The Sil-vinyl-acrylamide was similar to the Ref [2]. The detail process is as followings: 2.3 g of vinyl functionalized silica was added to 30 ml of methanol, ultrasonic, then 1.5g of acrylamide aqueous solution was added and stired at room temperature for 20 min and then the temperature was raised to 70 °C, 100 mg AIBN was also added and stirring the reaction for 6 h, and the centrifugation products were washed with water and methanol in order to obtain the Sil-vinyl-acrylamide.

The method for preparing another hydrophilic material (Silica@HTC) is involved:

The hydrothermal carbon-coated silica microspheres were produced by hydrothermal carbonization of cyclodextrin, which was inspired by previous literature [3].

Contents

1. Nuclear magnetic resonance hydrogen spectra of PEI reacting with epoxy silanization/chromopropyl trichlorosilane (**Figure S1)**

2. N_2 adsorption-desorption isotherms and BJH-adsorption pore size distribution of Sil-epoxy-PEI packing particles (a) and Sil-chloropropyl-PEI particles (b) (**Figure S2**).

3. Zeta potential assay of the Sil-epoxy-PEI packings (black line) and Sil-chloropropyl-PEI packings (red line) (**Figure S3**).

4. Repeatability assays of the Sil-epoxy-PEI packings and Sil-chloropropyl-PEI spheres (**Figure S4**).

5. Separation of benzylamine, p-phenylenediamine, o-phenylenediamine of the Sil-epoxy-PEI and Sil-chloropropyl-PEI packings. Mobile phase: ACN/water (90/10, v/v), flow rate: 1 ml/min (**Figure S5**).

6. Results of separation of saccharides from water extract of Euphorbia officinalis on Sil-vinyl-GSH column. Mobile phase gradient: A-water, B-ACN, gradient elution:0~30 min, 25%~50% A, 75%~50% B, 30~50 min, 50% A, 50% B, flow rate: 1 mL/min. detector: evaporative light scattering, tube temperature: 50 ℃ (**Figure S6**).

7. Results of separation of saccharides from water extract of Euphorbia officinalis on Sil-vinyl-acrylamide column. mobile phase gradient: A-water, B-ACN, gradient elution:0~30 min, 25%~50% A, 75%~50% B, 30~50 min, 50% A, 50% B, flow rate: 1 mL/min. detector: evaporative light scattering, tube temperature: 50 ℃ (**Figure S7**).

8. Results of separation of saccharides from water extract of Euphorbia officinalis on Silica@HTC column. mobile phase gradient: A-water, B-ACN, gradient elution:0~30 min, 25%~50% A, 75%~50% B, 30~50 min, 50% A, 50% B, flow rate: 1 mL/min. detector: evaporative light scattering, tube temperature: 50 ℃ (**Figure S8**)

As shown in Figure S1, PEI was reacted with epoxy silanizing/chromopropyl trichlorosilane separately, and the resulting compounds were analyzed by nuclear magnetic resonance spectroscopy. The results are as follows：

For the first, ^{1}H NMR (D_2O, 400 MHz, δ, ppm): δ =3.17 (s), 2.53 (t). The hydrogen with a chemical shift of 3.17 ppm is in a singlet state without splitting and the triple peak at 2.53 ppm splitting peaks were observed.

Secondly, ^{1}H NMR (D_2O, 400 MHz, δ, ppm): δ=0.05 (s), 2.73 (t). 2.73 ppm represents the splitting of the H bond between methyl and Cl (in the following picture) was also observed.

Usually the hydrogen that's in 2.3 to 2.90 and usually those hydrogens are coming out of this position, which is the polyethyleneimine itself.

3.17 ppm, that set of peaks. It's one of those carbons that the H shift bonded onto the carbon and/or oxygen atom after reacting of PEI with the epoxy monomer.

Especially 0.05 ppm, and actually it was the one shift when the chloropropyl reacted with PEI sample. Those carbon atoms that's the chloropropyl group between silicon atom and nitrogen that should really be the peak of a very high field.

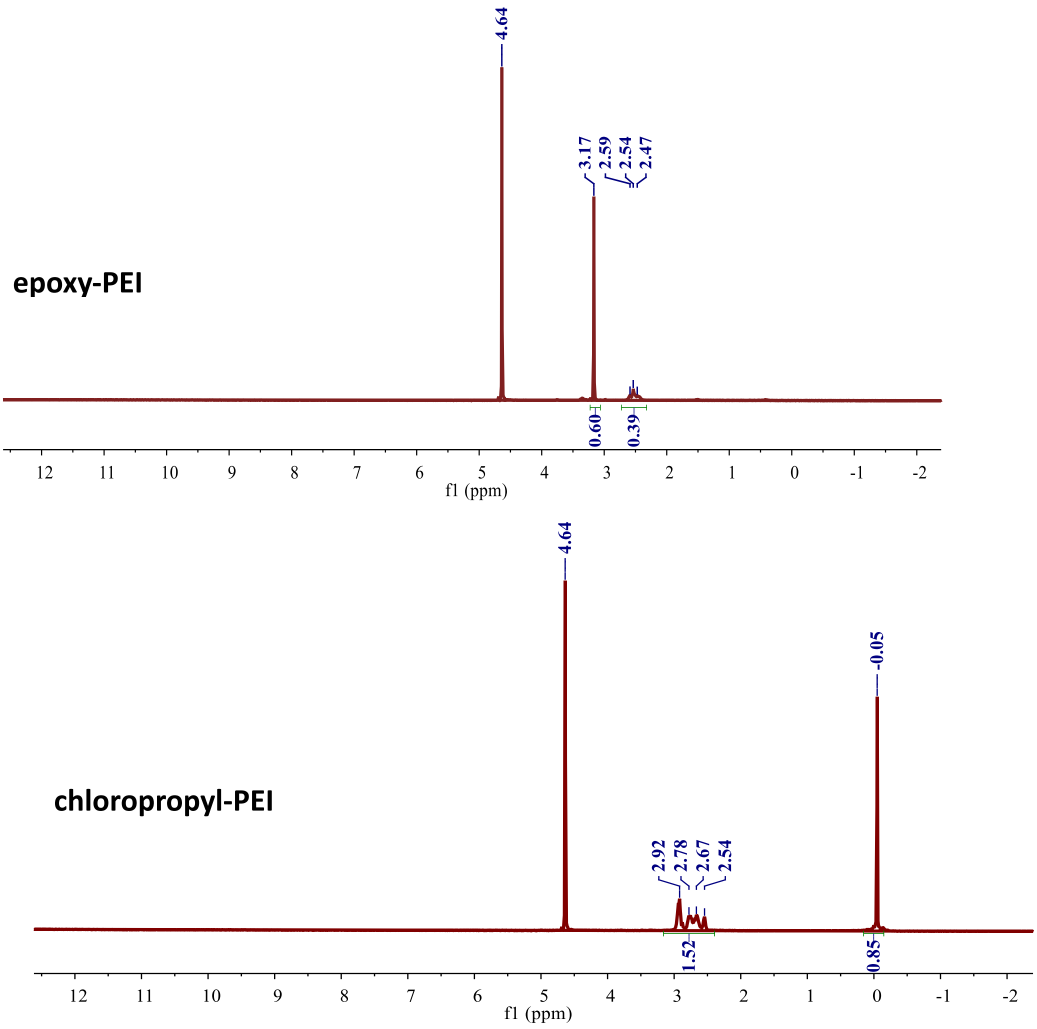

Figure S1. Nuclear magnetic resonance hydrogen spectra of PEI reacting with epoxy

silanization/chromopropyl trichlorosilane

As shown in Figure S2A and B, the N_2 adsorption–desorption isotherms with the Brunauer-Emmett-Teller (BET) equation and the pore size distribution with Barrett-Joyner-Halenda (BJH) equation manifested the changes of the surface area, pore volume and the pore diameter of the synthesized microspheres (Supporting information Table S1). In a contrast, the calculated pore diameter of the synthesized microspheres was ca.9.14 nm and 8.91 nm smaller than the 9.5 nm of the raw silica microspheres.

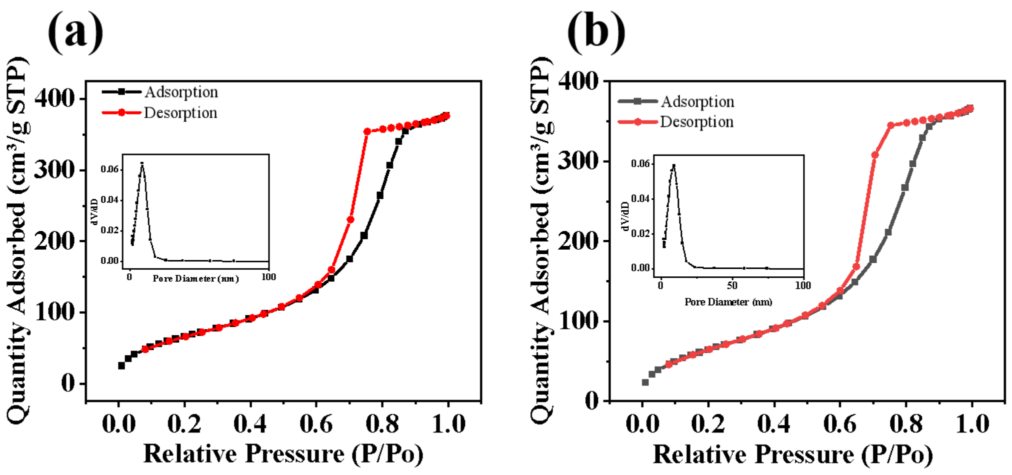

Figure S2. N_2 adsorption-desorption isotherms and BJH-adsorption pore size distribution of Sil-epoxy-PEI packing particles (a) and Sil-chloropropyl-PEI particles (b).

Table S1. Particles S_{BET}, Pore size and Pore volume of Sil-epoxy-PEI and Sil-chloropropyl-PEI

Materials	Particles S_{BET} (m^2g^{-1})	Pore size (nm)	Pore volume ^{c} (cm^3g^{-1})
silica	293.57	9.26	0.78
Sil-epoxy-PEI	254.92	9.14	0.58
Sil-Cl-PEI	253.99	8.91	0.56

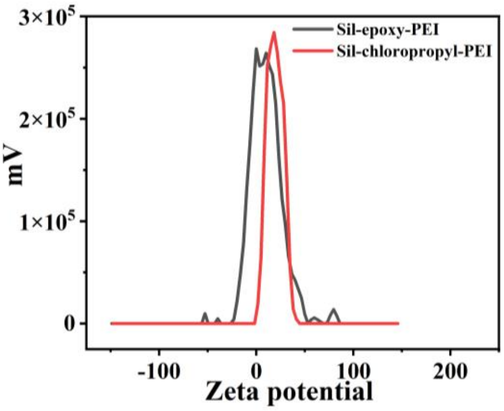

Figure S3. Zeta potential assay of the Sil-epoxy-PEI (black line) and Sil-chloropropyl-PEI packings (red line).

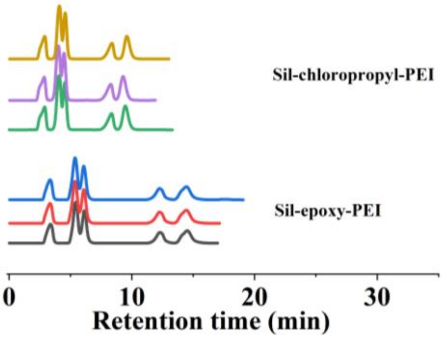

Figure S4. Repeatability assays of the Sil-epoxy-PEI packings and Sil-chloropropyl-PEI spheres.

Table S2. Retention time for repeatability assays of the Sil-epoxy-PEI and Sil- chloropropyl-PEI packings.

	Repeat time	Retention time	Repeat time	Retention time	Repeat time	Retention time
Sil-epoxy-PEI	1	3.33	2	3.37	3	3.36
		5.36		5.42		5.39
		6.07		6.12		6.10
		12.25		12.28		12.25
		14.44		14.52		14.52
Sil-chloropropyl-PEI	1	2.86	2	2.90	3	2.89
		4.00		4.09		4.07
		4.47		4.56		4.53
		8.28		8.37		8.35
		9.28		9.59		9.47

Table S3. Retention time and resolution of the four sugars at 65% ACN, 70% ACN, 75% ACN of the Sil-epoxy-PEI packings, Sil-chloropropyl-PEI packings and AMIDE-GEL 5.

		Sil-epoxy-PEI			Sil-chloropropyl-PEI			AMIDE-GEL 5		
ACN %		Retention time/min	Peak width	R (maltose and raffinose)	Retention time/min	Peak width	R (maltose and raffinose)	Retention time/min	Peak width	R (maltose, raffinose)
65%	glucose	4.50	0.36	3.53	3.27	0.47	1.3	3.51	0.29	2.47
	Maltose	5.98	0.47		4.07	0.69		4.38	0.44	
	raffinose	7.73	0.52		4.95	0.66		5.63	0.56	
	stachyose	11.73	0.79		6.75	1.09		8.24	0.36	
70%	glucose	5.86	0.46	6.06	3.92	0.35	2.72	4.27	0.40	3.69
	Maltose	8.86	0.64		5.40	0.36		5.93	0.61	
	raffinose	13.13	0.77		7.35	1.06		8.67	0.87	
	stachyose	23.65	1.20		11.74	0.05		15.08	0.60	
75%	glucose	7.19	0.58	16.49	5.09	0.31	45.53	5.63	0.54	12.13
	Maltose	13.36	0.82		8.27	0.17		9.16	0.37	
	raffinose	24.20	0.48		13.23	0.04		16.06	0.76	
	stachyose				25.60	0.08				

Table S4. Retention time of Morinda oligosaccharides of the Sil-epoxy-PEI packings and Sil-chloropropyl-PEI gel and AMIDE-GEL 5.

		3	4	5	6	7	8	9	10	11	12	13
Sil-epoxy-PEI	time/min	18.67	21.67	24.30	26.8	29.0	31.0	32.80	34.43	35.92	37.25	38.52
Sil-chloropropyl-PEI		13.39	16.52	19.30	22.4	24.7	26.80	28.54	30.33	31.86	/	/
AMIDE-GEL 5		13.63	16.16	18.40	20.70	22.70	24.60	26.28	27.82	29.23	30.54	31.77

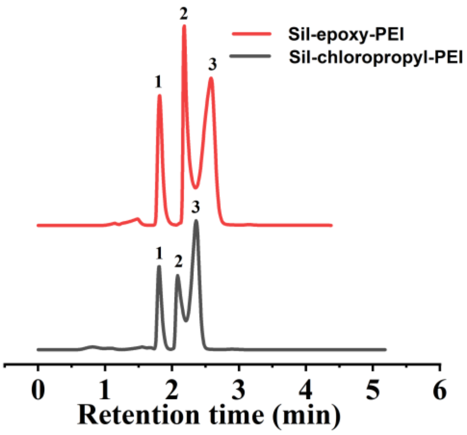

Figure S5. Separation of (1) benzylamine, (2) p-phenylenediamine, (3) o-phenylenediamine of the Sil-epoxy-PEI and Sil-chloropropyl-PEI packings. Mobile phase: ACN/water (90/10, v/v), flow rate: 1 ml/min.

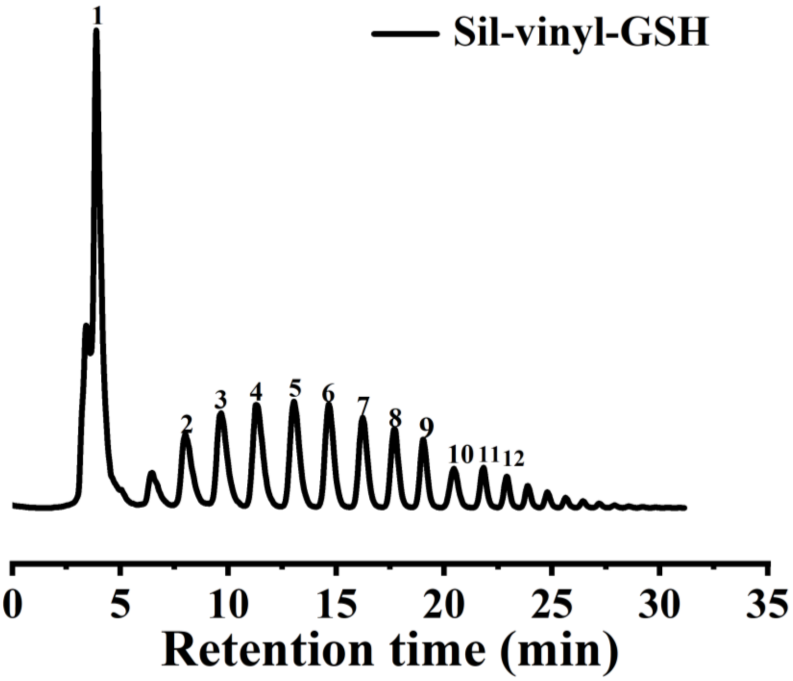

Figure S6. Results of separation of saccharides from water extract of Euphorbia officinalis on Sil-vinyl-GSH column. mobile phase gradient: A-water, B-ACN, gradient elution:0~30 min, 25%~50% A, 75%~50% B, 30~50 min, 50% A, 50% B, flow rate: 1 mL/min. detector: evaporative light scattering, tube temperature: 50 ℃.

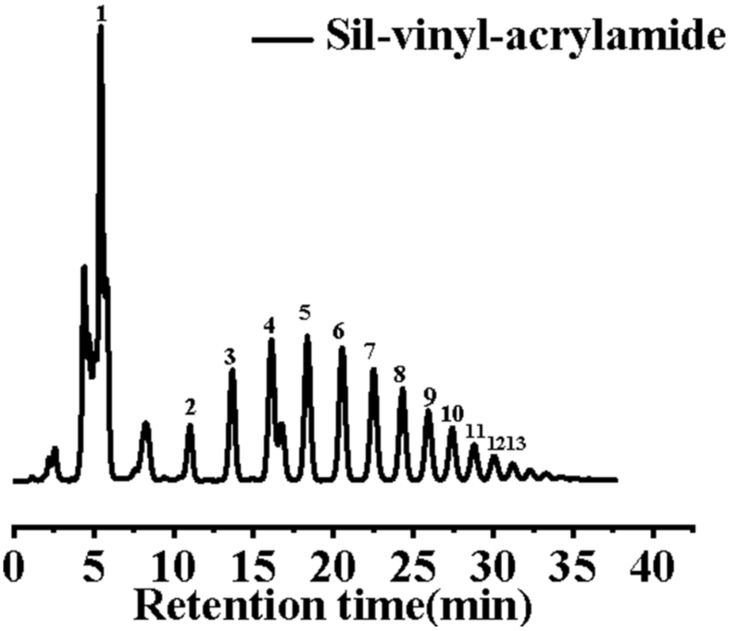

Figure S7. Results of separation of saccharides from water extract of Euphorbia officinalis on Sil-vinyl-acrylamide column. mobile phase gradient: A-water, B-ACN, gradient elution:0~30 min, 25%~50% A, 75%~50% B, 30~50 min, 50% A, 50% B, flow rate: 1 mL/min. detector: evaporative light scattering, tube temperature: 50 ℃.

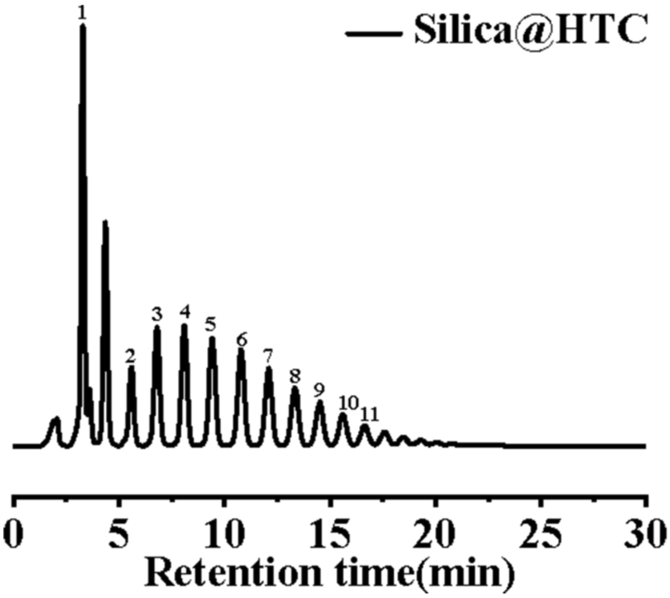

Figure S8. Results of separation of saccharides from water extract of Euphorbia officinalis on Silica@HTC column. mobile phase gradient: A-water, B-ACN, gradient elution:0~30 min, 25%~50% A, 75%~50% B, 30~50 min, 50% A, 50% B, flow rate: 1 mL/min. detector: evaporative light scattering, tube temperature: 50 ℃.

References

1. Shen A, Li X, Dong X, et al. Glutathione-based zwitterionic stationary phase for hydrophilic interaction/cation-exchange mixed-mode chromatography *Journal of Chromatography a*, **2013**, 1314: 63-69.
2. Wang X, Cui J, Zhou J, et al. Preparation of polyacrylamide hydrophilic stationary phases with adjustable performance *Journal of Chromatography A*, **2023**, 1702: 464065.

3. Zhao X, Liu S, Peng J, et al. Facile one-pot synthesized hydrothermal carbon from cyclodextrin: A stationary phase for hydrophilic interaction liquid chromatography *Journal of Chromatography A*, **2019**, 1585: 144-151.
